# Fast Compressed Sensing MRI Based on Complex Double-Density Dual-Tree Discrete Wavelet Transform

**DOI:** 10.1155/2017/9604178

**Published:** 2017-04-09

**Authors:** Shanshan Chen, Bensheng Qiu, Feng Zhao, Chao Li, Hongwei Du

**Affiliations:** ^1^Centers for Biomedical Engineering, University of Science and Technology of China, Hefei, Anhui 230027, China; ^2^School of Computer Science, University of Lincoln, Brayford Pool, Lincoln LN6 7TS, UK

## Abstract

Compressed sensing (CS) has been applied to accelerate magnetic resonance imaging (MRI) for many years. Due to the lack of translation invariance of the wavelet basis, undersampled MRI reconstruction based on discrete wavelet transform may result in serious artifacts. In this paper, we propose a CS-based reconstruction scheme, which combines complex double-density dual-tree discrete wavelet transform (CDDDT-DWT) with fast iterative shrinkage/soft thresholding algorithm (FISTA) to efficiently reduce such visual artifacts. The CDDDT-DWT has the characteristics of shift invariance, high degree, and a good directional selectivity. In addition, FISTA has an excellent convergence rate, and the design of FISTA is simple. Compared with conventional CS-based reconstruction methods, the experimental results demonstrate that this novel approach achieves higher peak signal-to-noise ratio (PSNR), larger signal-to-noise ratio (SNR), better structural similarity index (SSIM), and lower relative error.

## 1. Introduction

Magnetic resonance imaging (MRI) is a powerful noninvasive imaging modality, which is ubiquitously used in modern medical diagnosis [1]. MRI provides comparable spatial resolution with ultrasound and yields superior performance than CT in soft-tissue imaging. Nevertheless, the long scanning time limits its applications. Compressed sensing (CS) can exploit the sparsity of MR images in the transform domain and perfectly recover images from fewer measurements than those suggested by the traditional Nyquist sampling theory [2–4]. Furthermore, CS-MRI can reduce the number of samples, effectively shorten the scanning time, and then obtain successful recovery if two prerequisites are satisfied: (i) the raw imaging data must have a sparse representation in a known transform domain and (ii) the undersampling artifacts appear sufficiently incoherent in the sparsifying transform domain [3]. However, the quality of the reconstructed images is poor when the *k*-space data are highly undersampled and the representation is not sparse enough.

In recent years, a variety of techniques have been proposed to enhance the quality of MRI, which can be roughly classified into three categories [5]: incoherent undersampling pattern [6], sparse representation [7], and nonlinear reconstruction algorithms [8–11]. The first strategy (e.g., variable density random *k*-space sampling [6], spirals sampling [12], radial sampling [13], and Gaussian random sampling [14]) takes advantage of designing the *k*-space sampling pattern to shorten the sampling time, increase the imaging speed, and reduce the motion artifacts. However, aliasing artifacts may occur in these sampling methods. The high-frequency part contains less image information than the low-frequency part. Hence, by using undersampling patterns, the information about details is lost in the reconstructed images. Besides, the substantial aliasing artifacts appear incoherent. In case that the sampling ratio is extremely low, it is almost impossible to remove the significant aliasing artifacts from real signals [6, 8, 9]. For the second approach, it is essential to find a suitable sparsifying transform to recover images from highly undersampling *k*-space data. The discrete wavelet transform (DWT) is widely applied in CS-MRI, but it is sensitive to shift, lacks information about phase, and has poor directionality [15]. Wavelets cannot sparsely represent curves and may lead to visible artifacts. The contourlet sparse transform is another popular alternative that can efficiently capture the contour information. This transform exhibits superior performance in representing curves, but it may fail in representing singular points [16]. The stationary wavelet transform (SWT) can noticeably reduce pseudo-Gibbs artifacts [17]. Similar to DWT, SWT can only possess three spatial directions. Thus, when the original image involves rich directional information, the recovered images may become blurred. The complex double-density dual-tree discrete wavelet transform (CDDDT-DWT) has the characteristics of antialiasing properties and shift invariance and is approximate to continuous wavelet transform. Moreover, it has excellent directional selectivity that can better describe the direction of the original image [18, 19]. The third technique explores an effective nonlinear reconstruction algorithm to solve the optimization problem, which is usually a combination of least square fitting and *ℓ*_1_-norm regularization. These approaches, such as conjugate gradient [8], iterative shrinkage/soft thresholding algorithm (ISTA) [20], two-step ISTA (TwIST) [21, 22], and fast iterative shrinkage/soft thresholding algorithm (FISTA) [23], have been investigated intensively in the literature. However, each of them has limitations. For instance, the convergence speed of conjugate gradient is very slow due to the high time-complexity. ISTA is quite sensitive to the step size and its convergence speed may be rather slow especially when the measurement matrix is seriously ill-conditioned. For TwIST and FISTA, their estimates are not only dependent on the previous one, but also related to two or more previous estimates. Moreover, the global convergence rate of TwIST has not been thoroughly studied, while FISTA inspired by Nesterov's optimal algorithm [24] can be easily implemented and is sufficient to solve large-scale convex problems. It has been proved that the convergence rate of FISTA is *O*(1/*k*^2^), where *k* is the number of iterations.

To enhance the image reconstruction quality and reduce the reconstruction artifacts, in this paper, we propose a novel reconstruction scheme, which combines CDDDT-DWT with FISTA. Although dual-tree complex wavelet transform has also been exploited in the literature [25], its directional selectivity is inferior to CDDDT-DWT. It may suffer from artifacts as well, especially when the original image contains information in several directions. The CS-MRI combining with CDDDT-DWT was first introduced in [15]. In comparison with [15], the FISTA algorithm [23] with faster convergence rate was utilized to replace conventional conjugate gradient algorithm that costs more computational time.

The remainder of this paper is organized as follows.  Section 2 presents the new sparsity transform and briefly describes the basics of CS as well as the proposed FISTA-CDDDT method. The experimental results of the proposed approach and its comparison with other state-of-the-art techniques are illustrated in Section 3. In Section 4, the discussion for our algorithm is presented.  Section 5 concludes the paper.

## 2. Materials and Methods

### 2.1. Complex Double-Density Dual-Tree DWT

The CDDDT-DWT is an overcompleted discrete wavelet transform that combines double-density DWT [19] with dual-tree CWT [26]. It consists of two scale functions *φ*_*h*_(*t*) and *φ*_*g*_(*t*) and four distinct wavelets *φ*_*h*,*i*_(*t*) and *φ*_*g*,*i*_(*t*)(*i* = 1,2), where *φ*_*h*,1_(*t*) is an offset from *φ*_*h*,2_(*t*) by one-half and *φ*_*g*,1_(*t*) is an offset from *φ*_*g*,2_(*t*) by one-half. One pair of wavelets *φ*_*h*,*i*_(*t*) and *φ*_*g*,*i*_(*t*)(*i* = 1,2) form an approximation of the Hilbert transform pair [18]; namely, *φ*_*g*,1_(*t*) ≈ *ℋ*{*φ*_*h*,1_(*t*)}, *φ*_*g*,2_(*t*) ≈ *ℋ*{*φ*_*h*,2_(*t*)}.

**Figure 1:**
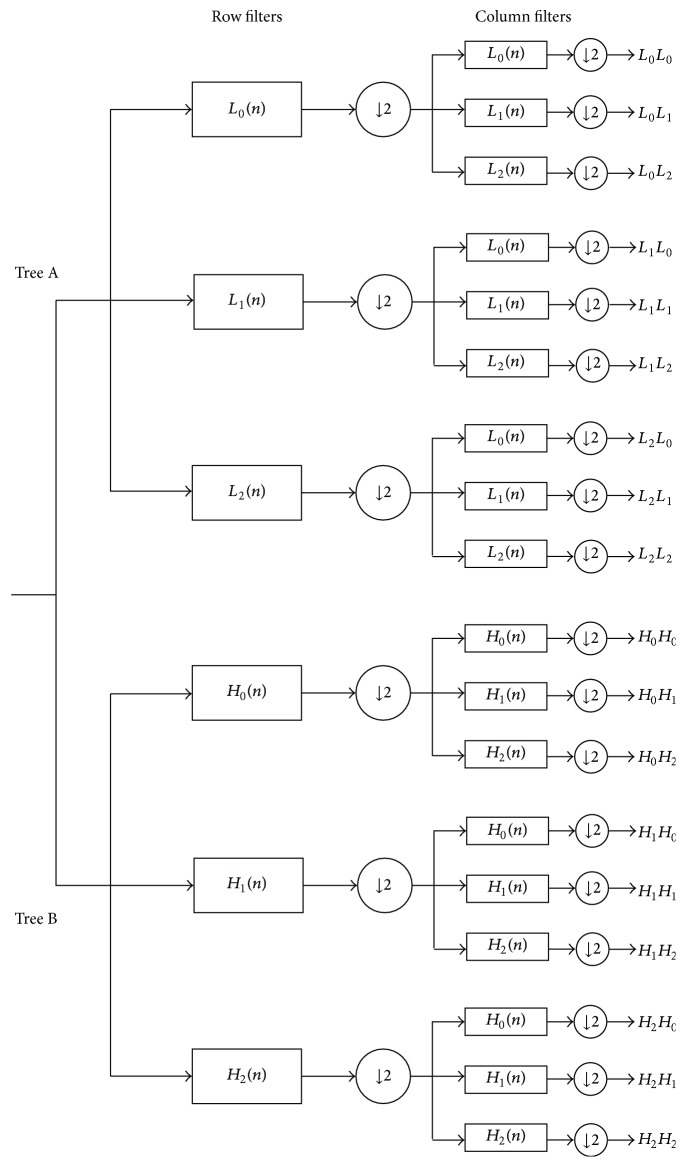
The filter bank structure for real 2D double-density dual-tree DWT.

Two-dimensional (2D) double-density dual-tree DWT includes 2D real double-density dual-tree DWT and 2D complex double-density dual-tree DWT. The former is constructed from two oversampled 2D double-density DWT in parallel, which is redundant by a factor of two.  Figure 1 shows its filter bank structure, where the row and the column filters produce two low-frequency subbands (i.e., *L*_0_*L*_0_, *H*_0_*H*_0_) and 16 high-frequency subbands (i.e., *L*_0_*L*_1_, *L*_0_*L*_2_, *L*_1_*L*_0_, *L*_1_*L*_1_, *L*_1_*L*_2_, *L*_2_*L*_0_, *L*_2_*L*_1_, *L*_2_*L*_2_, *H*_0_*H*_1_, *H*_0_*H*_2_, *H*_1_*H*_0_, *H*_1_*H*_1_, *H*_1_*H*_2_, *H*_2_*H*_0_, *H*_2_*H*_1_, and *H*_2_*H*_2_) to describe the details of the recovered image.

**Figure 2:**
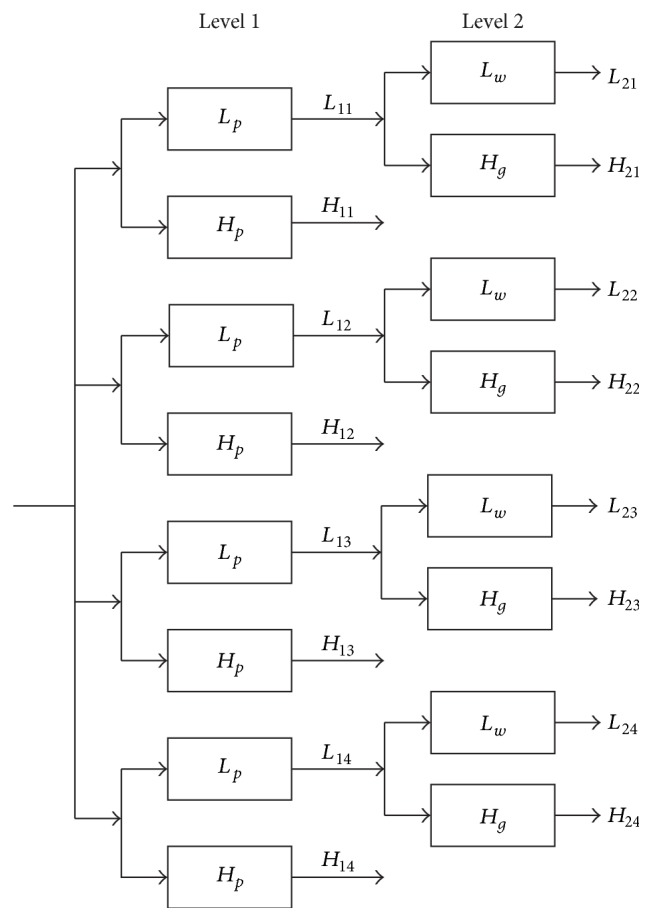
The two levels of 2D complex double-density dual-tree DWT.

The latter is formed by utilizing four oversampled 2D double-density DWT in parallel to the input image. The filter bank structure of this transform can be obtained by extending the one illustrated in Figure 1. As shown in Figure 2, *L*_*p*_ and *H*_*p*_ make up the filter banks of the first-level decomposition, where *L*_*p*_ represents a scale filter and *H*_*p*_ depicts eight wavelet filters, while *L*_*w*_ and *H*_*g*_ denote the filter bank structures of the second-level decomposition. Each level generates four low-frequency subbands (*L*_*ij*_, *i* = level, *j* = 1,…, 4) and 32 high-frequency subbands (*H*_*ij*_, *i* = level, *j* = 1,…, 4) through 2D CDDDT-DWT transform. Similar to other wavelet transforms, the redundant transform is achieved by recursively applying low-frequency subbands to complete the decomposition of each level. For each pair of subbands, CDDDT-DWT takes their summation and difference to produce the 32 oriented wavelets, describing a total of 16 main directions. Besides, each main direction contains two distinct wavelet representations, which indicate the real part of a complex-valued 2D wavelet function and the imaginary part, respectively [27].

### 2.2. Proposed FISTA-CDDDT Algorithm

The CS-MRI image reconstruction problem is defined as follows:

(1) minx ϕxs.t. Fux−y22≤ξ,

where *x* denotes the fully sampled image, *y* is the *k*-space data acquired from a MR scanner, and *ξ* (*ξ* > 0) is a parameter appropriately chosen based on the noise level, which controls the difference between the object image and the reconstructed one. *F*_*u*_ is the undersampled Fourier transform in MRI. *ϕ*(·) is called the regularization function in the transform domain, which is generally nonsmooth. This optimization problem can be potentially solved by total variation- (TV-) based approaches [10], but we will not discuss them in this paper. Here, the constrained optimization problem in (1) can be transformed into the following unconstrained one by using Lagrangian function:

(2) $$\begin{array}{c} \hat{x}=arg\;\underset{x}{min}\frac{1}{2}{\left\|\;F_{u}x-y\;\right\|}_{2}^{2}+\tau \phi \left(\;x\;\right), \end{array}$$

where *τ* is a positive regularization parameter. To solve (2), *ℓ*_0_ “norm” (‖*x*‖_0_ = |{*i* : ≠0}|) is chosen as the regularization function. *ϕ*(*x*) = ‖*x*‖_0_ especially provides the simplest way to enforce the sparsity:

(3) x^=arg minx12Fux−y22+τΦix0,i=1,2,…,16,

where *x* can be sparsely represented in this selected domain Φ. Here, Φ_*i*_ (*i* = 1,2,…, 16) is the 16 high-frequency subbands of CDDDT-DWT, which serves as a new sparse basis. However, the solution of (3) is a NP hard problem, which means that a solution within polynomial time is not guaranteed [11].

As an alternative formulation, applying *ℓ*_1_-norm directly to the regularization function produces the result formally defined as

(4) x^=arg minx12Fux−y22+τΦix1,i=1,2,…,16.

Since *ℓ*_1_-norm is nonsmooth and convex, (4) can be considered as the convex relaxation of (3) to effectively solve the quadratic convex problem. In the underdetermined problem (4), *f*(*x*) = (1/2)‖*F*_*u*_*x* − *y*‖_2_^2^ represents the quadratic term, which is a convex function with Lipschitz continuous gradient, and *g*(*x*) = *τ*‖Φ_*i*_*x*‖_1_, *i* = 1,2,…, 16 is a nonsmooth convex regularizer.

The FISTA algorithm is applied to solve the optimization problem of (4). For a given point *z*_*k*_, we can get the gradient of *f*(*x*) at *z*_*k*_ by

(5) ∇fzk=FuTFuzk−y,xg=zk−ρFuTFuzk−y,

where *F*_*u*_^*T*^(*F*_*u*_*z*_*k*_ − *y*) denotes the gradient of *f*(*x*) at the given point *z*_*k*_, which is a specific combination of the previous estimate values *x*_*k*_, *x*_*k*−1_. The original FISTA based on wavelet transform has been well studied in the literature with a backtracking step size or a constant step size *ρ*, both of which can provide an improved global convergence rate of *O*(1/*k*^2^) [23]. For simplicity, most algorithms adopt a constant step size in the direction of the negative gradient of the convex function.

Applying the sparsity transform CDDDT-DWT to a local optimal image *x*_*g*_, we can get

(6) $$\begin{array}{c} w_{h}=\tau {\left\|\;\Phi_{i}x_{g}\;\right\|}_{1},\;i=1,2,\ldots ,16, \end{array}$$

where *w*_*h*_ is the new wavelet coefficients, which can be adjusted by the proximal forward-backward iterative scheme [28] to catch the accurate coefficients. Although *ℓ*_1_-norm is nonsmooth, it is separable and CDDDT-DWT has the characteristics of tight frame. It is known that the shrinkage thresholding function with threshold *T* is utilized to obtain the modified wavelet coefficients *w*_*h*_′:

(7) shrinkwh,T=whwh∗maxwh−T,0,wh′=shrinkwh,T.

The recovered image *x*_*k*_ is updated by

(8) $$\begin{array}{c} x_{k}=\Phi_{i}^{-1}w_{h}^{\prime },\;i=1,2,\ldots ,16. \end{array}$$

In (8), the inverse CDDDT-DWT (Φ_*i*_^−1^,  *i* = 1,2,…, 16) is applied by the synthesis filter bank structure, constituted by inverse order of the analysis filter bank [27]. *β* is a threshold relaxation factor to adjust *T*, which optimizes *T* and reduces the calculation time. When the stop condition *T* ≤ *ϵ* is satisfied, we obtain the optimal solution of (4).

The proposed algorithm combining the complex double-density dual-tree and fast iterative shrinkage thresholding algorithm (FISTA-CDDDT) for solving (4) is depicted as in Algorithm 1.

## 3. Experiments

### 3.1. Experimental Setup

**Figure 3:**
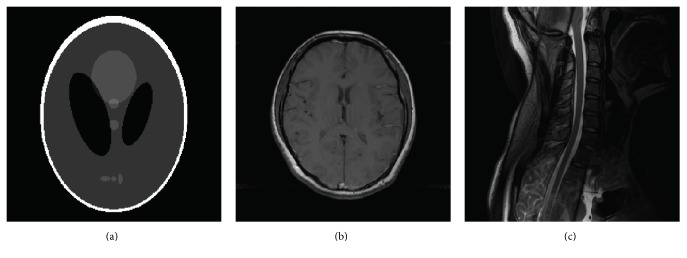
The MR images. (a) Shepp-Logan phantom, (b) axial brain, and (c) spine.

To evaluate the performance of the proposed reconstruction algorithm, we implement the complex double-density dual-tree wavelet and conventional wavelet using the software in [27, 29]. The experiments are conducted on three typical MR datasets: Shepp-Logan phantom [17], axial brain MR data, and spine MR data, as shown in Figures 3(a)–3(c). The first Shepp-Logan phantom is piecewise smooth and strictly sparse, which involves the directional curves and thus can be used for testing the proposed algorithm. The complex *k*-space data of the axial brain are acquired by a 3T GE MR750 scanner using fast spin echo sequence (TR/TE = 500/12.9 ms, field of view = 240 × 240 mm, and slice thickness = 5 mm). The spine MR data are a fully sampled *k*-space data obtained by a 3T GE MR750 system with FRFSE sequence (TR/TE = 2500/110 ms, field of view = 240 × 240 mm). For the sake of brevity, the size of all testing images is scaled to 256 × 256. All experiments are performed using MATLAB 2014b on a desktop computer with a 3.2 GHz Intel core i5-4460 CPU.

**Figure 4:**
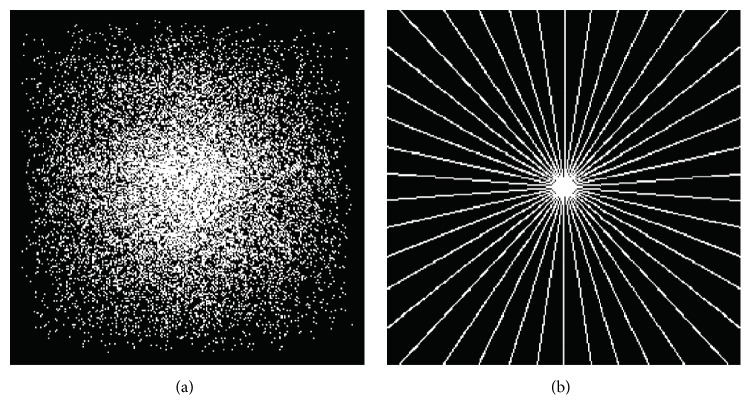
The undersampling patterns. (a) Gaussian random sampling at a sampling ratio of 20% and (b) radial sampling at a sampling ratio of 9%.

Gaussian random *k*-space pattern and radial undersampling pattern are used to undersample the fully sampled *k*-space raw data. For most of MR images, the *k*-space signal with a large magnitude is generally localized in the central part.  Figure 4(a) shows a Gaussian random sampling pattern, which randomly collects more low-frequency signals in the central region of *k*-space and less high-frequency signals in the peripheral region of *k*-space. The radial undersampling pattern displayed in Figure 4(b) contains 22 radial lines with a sampling ratio of 9% in the Fourier transform domain. The sampling ratio, defined as the number of sampled points divided by the total size of original image, depends on the number of radial lines. The more the radial lines are, the higher the sampling ratio will be. It is worth noting that all the experiments can use the spiral or Cartesian sampling pattern as well.

### 3.2. Experimental Methods

In this work, FISTA based on three different sparsity transforms is utilized to solve the optimization problem of (4). These three techniques are implemented under the same conditions. The first method combines the discrete wavelet transform with FISTA (FISTA-DWT), the second algorithm integrates the complex dual-tree wavelet transform with FISTA (FISTA-CDT), and the third approach incorporates the complex double-density dual-tree wavelet transform with FISTA (FISTA-CDDDT). For both the simulation and experiments on in vivo data, FISTA-DWT uses a Daubechies wavelet frame with four decomposition levels as a sparsity basis. FISTA-CDT utilizes a biorthogonal Daubechies wavelet with the 9/7 filters in the first stage and then exploits the Q-filter by Kingsbury in the second stage [25]. FISTA-CDDDT applies the finite impulse response (FIR) to perfectly reconstruct the filter banks.

We first conduct the experiment on the Shepp-Logan phantom image shown in Figure 3(a). The optimal values are experimentally set and all the three methods terminate after 100 iterations with 20% undersampling *k*-space data. In the axial brain experiment, we set the optimal parameters *ϵ* = 0.001, *ρ* = 1, *β* = 0.9, and *T* = 0.0095, while parameters in the spine experiment are set as *ϵ* = 0.0001, *ρ* = 1, *β* = 0.9, and *T* = 0.01. For different testing datasets, the tuning parameter *τ* is set to different values and the total number of iterations in all the cases is set to 120. Additionally, in both simulation and experiments on in vivo data, we add Gaussian white noise to simulate a realistic environment. For simplicity, the same standard derivation *σ* = 0.01 is used.

The peak signal-to-noise ratio (PSNR), signal-to-noise ratio (SNR), structural similarity (SSIM) index, and relative error (Rel.Err) are used to evaluate the FISTA-CDDDT recovery performance. The PSNR is calculated using the following equation:

(9) $$\begin{array}{c} PSNR=20\;\log_{10}\frac{MAX}{\sqrt{\frac{1}{MN}\sum_{i=0}^{M}\sum_{j=0}^{N}{\left(x\left(i,j\right)-\hat{x}\left(i,j\right)\right)}^{2}}}, \end{array}$$

where *x* denotes images reconstructed from fully sampled data, *M* and *N* are the number of rows and columns in the input image, respectively. MAX means the maximum possible pixel value in the input image data, and $\hat{x}$ is the reconstructed image.

The SNR is defined as

(10) $$\begin{array}{c} SNR=10\;\log_{10}\frac{\sum_{i=0}^{M}\sum_{j=0}^{N}x{\left(i,j\right)}^{2}}{\sum_{i=0}^{M}\sum_{j=0}^{N}{\left(x\left(i,j\right)-\hat{x}\left(i,j\right)\right)}^{2}}. \end{array}$$

The definition of the SSIM index is given by

(11) SSIMp,q=2μpμq+c12θpθq+c22θpq+c3μp2+μq2+c1θp2+θq2+c2θpθq+c3,

where *p* and *q* are the various local windows from the same local window in the two different images to be compared. *μ*_*p*_ and *μ*_*q*_ are the mean of *p* and *q*, respectively, while *θ*_*p*_ and *θ*_*q*_ represent their variance. *θ*_*pq*_ is the covariance of *p* and *q*. *c*_1_, *c*_2_, and *c*_3_ are three variables used to increase the stability of the results. Both SSIM index and SNR have the same criterion as PSNR; that is, the reconstruction quality is directly proportional to the value of the metrics.

The Rel.Err is defined as

(12) $$\begin{array}{c} Rel.Err=\frac{{\left\|x-\hat{x}\right\|}_{2}}{{\left\|x\right\|}_{2}}\times 100\%. \end{array}$$

A smaller Rel.Err indicates a higher similarity between the original image and the reconstructed one.

### 3.3. Experimental Results

Note that, for all the figures in this part, various approaches are labeled by different colors below the images. The green dotted lines mean FISTA-DWT, the pink dotted lines denote FISTA-CDT, and the blue lines represent the proposed FISTA-CDDDT.

**Figure 5:**
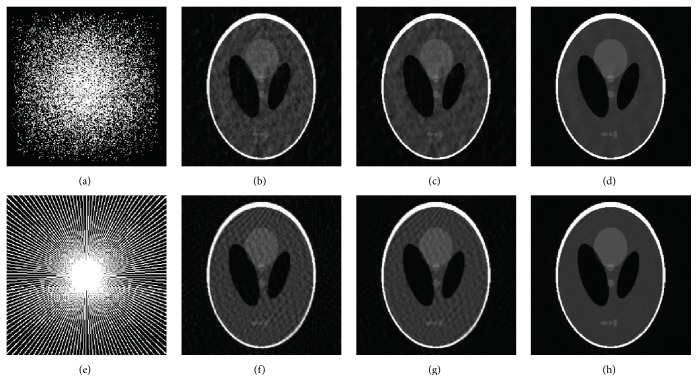
Reconstructed results of Shepp-Logan phantom images using Gaussian random mask (a–d) and radial mask (e–h) with a 20% sampling ratio among different approaches. (a) Gaussian random sampling mask, (b) and (f) FISTA-DWT, (c) and (g) FISTA-CDT, (d) and (h) FISTA-CDDDT, and (e) radial sampling mask.

Figure 5 gives the reconstructions by FISTA-DWT, FISTA-CDT, and FISTA-CDDDT using different sampling schemes at the same sampling ratio. The Gaussian random sampling mask shown in Figure 5(a) is applied on Shepp-Logan phantom. According to the reconstruction results presented in Figure 5, it can be seen that the image produced by FISTA-DWT contains serious artifacts due to undersampling and the one recovered by FISTA-CDT shows visible artifacts which are visually better than FISTA-DWT, whereas the artifacts in FISTA-CDDDT recovery are much less noticeable than FISTA-DWT and FISTA-CDT under the same conditions. Using the radial sampling mask illustrated in Figure 5(e), both FISTA-DWT and FISTA-CDT have the streaking artifacts, while the image recovered by the proposed method is the most similar one to the original image with no streaking artifacts. These streaking artifacts may be caused by low sampling ratio. Under such a sampling ratio, classic *ℓ*_1_-norm based CS techniques may result in poor performance with substantial artifacts. Consequently, CS methods cannot guarantee the quality of the reconstructed image at low sampling ratios.

**Figure 6:**
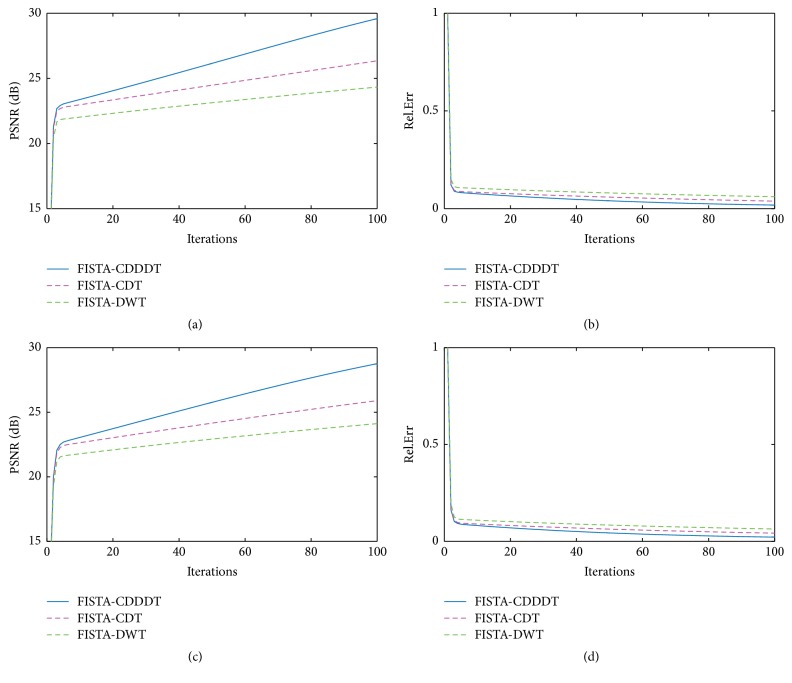
The comparison results among three different MR reconstruction algorithms using Gaussian random mask (a and b) and radial mask (c and d) at a 20% sampling ratio using Shepp-Logan phantom image. (a) and (c) PSNR versus iterations; (b) and (d) Rel.Err versus iterations.

Figure 6 illustrates that the proposed algorithm yields the best result with the highest PSNR and the lowest Rel.Err, where (a) and (c) describe the change of PSNR with the increasing number of iterations. All the three methods based on FISTA reconstruction have the same convergence rate. Since CDDDT-DWT adopts more wavelets and performs better in directional selectivity, the image reconstructed by this novel algorithm is much better than those by FISTA-CDT and FISTA-DWT, regardless of the adopted sampling patterns. Figures 6(b)–6(d) demonstrate that FISTA-CDDDT has less reconstruction errors than FISTA-CDT and FISTA-DWT.

**Figure 7:**
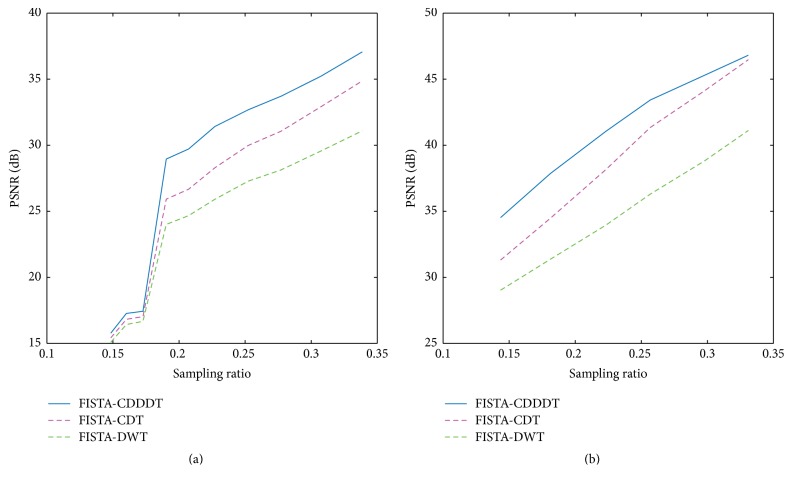
Comparisons among different approaches at different sampling ratios. (a) Gaussian random sampling and (b) radial sampling using a Shepp-Logan phantom image.

For further analysis, the PSNRs of the reconstructed images using different methods are plotted at various sampling ratios. It is clear from Figure 7 that, with the increase of the sampling ratio, the PSNR of all algorithms grows as well. Compared with traditional wavelet, the improvement of PNSR is approximately 6 dB applying radial sampling scheme. Therefore, the proposed scheme outperforms the other two in simulation.

**Figure 8:**
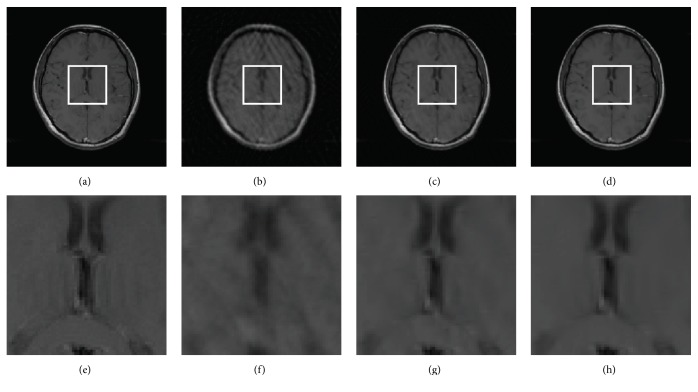
Reconstructed images using 20% radial sampling. (a) An axial brain image, (b) FISTA-DWT, (c) FISTA-CDT, (d) FISTA-CDDDT, and (e)–(h) magnified images of the regions marked by white rectangles in (a)–(d), respectively.

**Figure 9:**
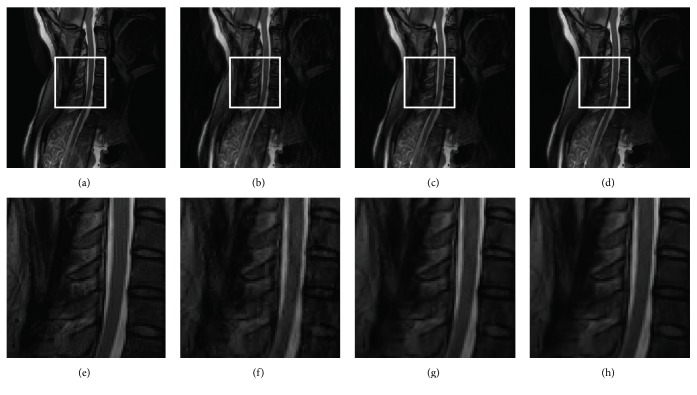
Reconstructed images using 20% Gaussian random sampling. (a) A spine image, (b) FISTA-DWT, (c) FISTA-CDT, (d) FISTA-CDDDT, and (e)–(h) magnified images of the regions marked by white rectangles in (a)–(d), respectively.

Figures 8 and 9 present all the reconstruction results of FISTA-DWT, FISTA-CDT, and the proposed FISTA-CDDDT. Additionally, images in the first row are recovered by using the same sampling mask and images in the second row are magnified images of the marked regions in the first row. Two imaging cases (axial brain image and spine MR image) are compared with each other at a 20% sampling ratio.  Figure 8(a) especially is reconstructed from full *k*-space data. The *k*-space data from each coil are reconstructed separately, and the final image is generated by the sum-of-square method [30].

Note that the PSNRs of reconstructed axial brain images using radial sampling mask by FISTA-DWT, FISTA-CDT, and FISTA-CDDDT are 33.99 dB, 37.58 dB, and 38.87 dB, respectively. The magnified images are shown in Figures 8(e)–8(h). From these figures, we can see that the brain structure in the local area becomes more and more distinct. The significant artifacts existing in Figure 8(b) may be caused by imperfect filter bank in the traditional wavelet. The synthesis and analysis filter banks adopted by our FISTA-CDDDT are more appropriate to obtain the curve details, especially in curve processing. The spine experiments (see Figure 9) demonstrate very similar results to the axial brain. Once again, this proves that the proposed method is more accurate and effective.

Table 1 gives the SNR, Rel.Err, and SSIM index for the reconstruction on an axial brain MR image at different radial sampling ratios with *σ* = 0.01. These results further demonstrate that the proposed FISTA-CDDDT is superior to the other two methods, because it exhibits the highest SNR, best SSIM index, and lowest Rel.Err.

**Figure 10:**
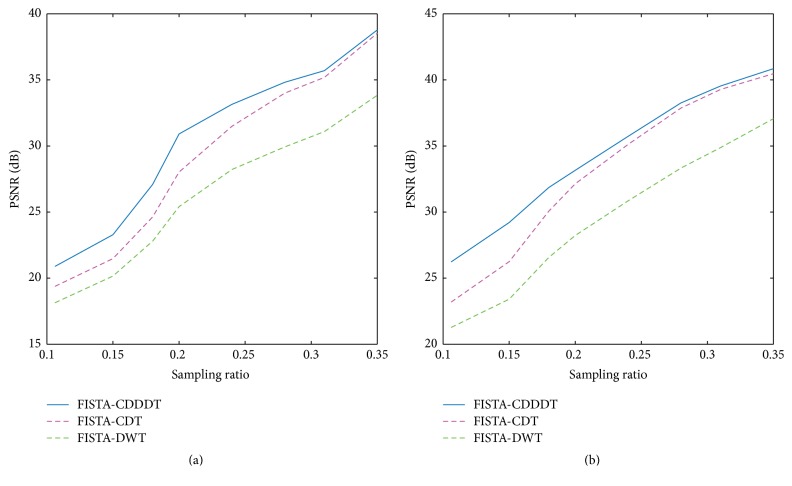
The comparison of PSNR versus sampling ratio among three different MR reconstruction algorithms using (a) Gaussian random mask and (b) radial mask on an axial brain image.

**Figure 11:**
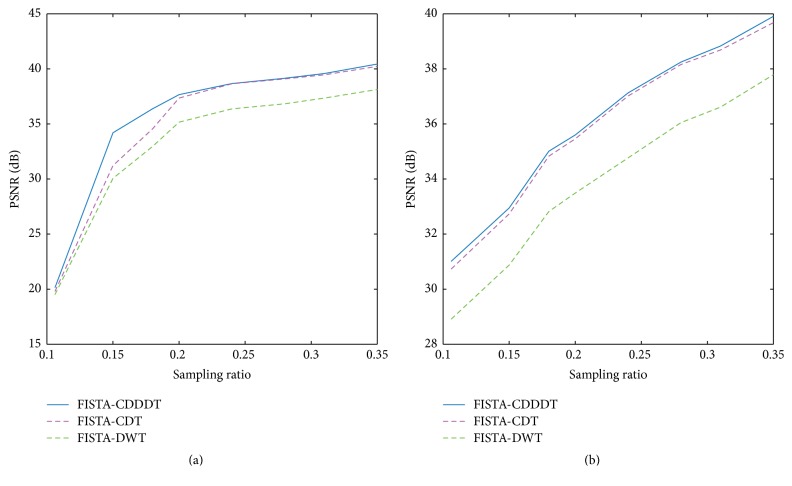
The comparison of PSNR versus sampling ratio among three different MR reconstruction algorithms using (a) Gaussian random mask and (b) radial mask on a spine image.

Figures 10 and 11 show the PSNR of the reconstructed images by FISTA-DWT, FISTA-CDT, and FISTA-CDDDT. In most cases, the proposed method has better performance than the other two approaches. However, when the Gaussian random sampling ratio is lower than 15%, the PSNR of our method is slightly higher than the other two. This is because when the sampling ratio is very low, the useful information about the main feature of the image is missing. The different evaluation criteria presented in Table 2 indicate that our method and FISTA-CDT are more effective than FISTA-DWT for performing reconstruction on a spine image at different sampling ratios. As the tissue structure of the cervical spine is too complicated, there is no obvious difference between the proposed algorithm and FISTA-CDT.

## 4. Discussion

Considering the superiority of CDDDT-DWT in preserving edges and maintaining higher directional selectivity, the proposed reconstruction approach combines the CDDDT-DWT with FISTA to produce better recovery results with a faster convergence rate. Although the ISTA and TwIST can be integrated with CDDDT-DWT as well, both of them were designed for simple regularization problems. Besides, they have some drawbacks that cannot be ignored. ISTA based on the operator-splitting strategy is a promising method, which has been successfully used in signal recovery. However, it belongs to the first-order algorithm that converges quite slow. As a variant of ISTA, TwIST is also an iterative thresholding algorithm, which is not guaranteed to converge globally. In contrast, FISTA has a faster convergence rate and better reconstruction accuracy, as proved in [23].

It is worth noting that Zhu et al. [15] designed an improved compressed sensing MRI algorithm (iCS), a variant of nonlinear conjugate gradient descent approach, to minimize the traditional CS model in which the image should be sparse in both the total variation and the specific CDDDT-DWT transform at the same time. The absolute value in iCS is approximated by a smooth function. In addition, the searching step size of backtracking line-search in iCS was set as 5, which may be too large, leading to an inexact solution and more computational time. Unlike iCS associated with composite regularization, the simple optimization model with *l*_1_-norm regularization is studied in this work. Furthermore, FISTA-CDDDT first uses proximal forward-back optimization to approximate the linearized function *f*(*x*), then applies shrinkage thresholding function to solve the minimization problem due to the separable characteristics of *l*_1_-norm, and finally adopts the specific linear combination of *x*_*k*_ and *x*_*k*−1_ to smartly select the search points. Besides, the threshold relaxation technique can further reduce the computational cost. Consequently, our method can gain a more accurate solution with a dramatically improved complexity of *O*(1/*k*^2^).

## 5. Conclusion

In this paper, we develop a new image reconstruction method for CS-MRI based on complex double-density dual-tree wavelet transform. The filter bank structure of the CDDDT-DWT is explored. This novel approach has been applied to Shepp-Logan phantom and axial brain and spine image reconstruction and compared with two popular methods, namely, FISTA-DWT and FISTA-CDT. The reconstructed results demonstrate that our scheme improves the PSNR and SNR as well as SSIM index and reduces the reconstructed artifacts significantly. In both simulation and experiments on in vivo data, we use the FISTA as the reconstruction algorithm. However, it can only solve the unconstrained minimization problems. An algorithm that can solve both unconstrained and constrained convex optimization problems will be studied in the future work.

## Figures and Tables

**Algorithm 1:**
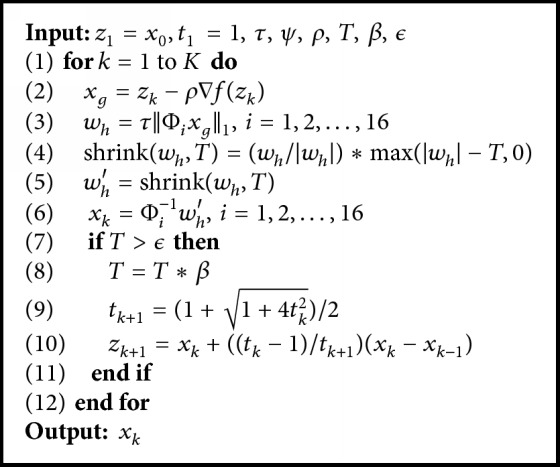
FISTA-CDDDT.

**Table 1:** Numerical results for an axial brain MR image by different reconstructed methods using radial sampling mask with *σ* = 0.01.

Sampling ratio	Algorithms	SNR (dB)	Rel.Err (%)	SSIM
15%	FISTA-DWT	14.51	4.99	0.7461
	FISTA-CDT	17.26	3.76	0.8393
	FISTA-CDDDT	20.26	3.19	0.9328

18%	FISTA-DWT	17.68	3.28	0.8409
	FISTA-CDT	21.15	2.41	0.9246
	FISTA-CDDDT	22.74	2.17	0.9558

20%	FISTA-DWT	19.84	2.61	0.8703
	FISTA-CDT	22.53	1.88	0.9427
	FISTA-CDDDT	23.82	1.76	0.9610

25%	FISTA-DWT	20.94	1.76	0.9067
	FISTA-CDT	24.37	1.30	0.9544
	FISTA-CDDDT	25.17	1.25	0.9651

28%	FISTA-DWT	22.35	1.36	0.9242
	FISTA-CDT	25.42	1.00	0.9600
	FISTA-CDDDT	26.01	0.99	0.9675

**Table 2:** Numerical results for a spine MR image using different reconstructed methods employing Gaussian random sampling mask with *σ* = 0.01.

Sampling ratio	Algorithms	SNR (dB)	Rel.Err (%)	SSIM
15%	FISTA-DWT	13.19	11.77	0.7555
	FISTA-CDT	14.35	11.55	0.7955
	FISTA-CDDDT	17.32	8.34	0.8694

18%	FISTA-DWT	16.08	7.48	0.8349
	FISTA-CDT	17.68	7.26	0.8656
	FISTA-CDDDT	19.51	3.57	0.9170

20%	FISTA-DWT	18.28	3.07	0.8937
	FISTA-CDT	20.48	2.43	0.9301
	FISTA-CDDDT	20.78	2.40	0.9435

25%	FISTA-DWT	19.49	2.30	0.9196
	FISTA-CDT	21.78	1.88	0.9520
	FISTA-CDDDT	21.80	1.81	0.9599

28%	FISTA-DWT	19.05	2.21	0.9262
	FISTA-CDT	22.22	1.90	0.9551
	FISTA-CDDDT	22.27	1.76	0.9262
